# KNDC1 knockdown protects human umbilical vein endothelial cells from senescence

**DOI:** 10.3892/mmr.2014.2201

**Published:** 2014-04-30

**Authors:** CHUNYAN ZHANG, YONG-ZHAN ZHEN, YA-JUN LIN, JIANG LIU, JIE WEI, RONG XU, GANG HU

**Affiliations:** 1 Radiologic Department, Beijing Shijitan Hospital Affiliated of Capital Medical University, Beijing 100038, P.R. China; 2 Department of Histology and Embryology, Basic Medical College of Hebei United University, Tangshan, Hebei 063000, P.R. China; 3 Key Laboratory of Geriatrics, Beijing Hospital and Beijing Institute of Geriatrics, Ministry of Health, Beijing 100730, P.R. China; 4 Department of Endocrinology, The Third Hospital of Nanchang City, Nanchang, Jiangxi 330009, P.R. China

**Keywords:** kinase noncatalytic C-lobe domain containing 1, knockdown, human umbilical vein endothelial cells, cellular senescence

## Abstract

KNDC1 (kinase noncatalytic C-lobe domain containing 1), a brain-specific Ras guanine nucleotide exchange factor, controls the negative regulation of neuronal dendrite growth. However, the effect of KNDC1 on cellular senescence remains to be elucidated. The present study investigated the impact of KNDC1 knockdown on human endothelial cell senescence and the mechanisms underlying this effect. Human umbilical vein endothelial cells (HUVECs) cultured *in vitro* were used as a model of biological aging. Senescence-associated β-galactosidase staining was used to detect cellular senescence and flow cytometry was employed to determine cell cycle progression. Quantitative polymerase chain reaction (qPCR) and western blot analysis were utilized to investigate mRNA transcription and protein expression. In the HUVECs, a senescence-like phenotypes developed with increasing passage number *in vitro*, which were associated with a progressive increase in the transcription and expression of KNDC1. KNDC1 knockdown promoted cell proliferation and partially reversed cellular senescence and cell cycle arrest in the G0/G1 phase in aging HUVECs. Investigations into the mechanism underlying this effect demonstrated that KNDC1 knockdown promoted HUVEC proliferation via the extracellular signal-regulated kinase signaling pathway and delayed HUVEC senescence by inhibiting the p53-p21-p16 transduction cascade. In addition, the promotion of the capillary tube network formation and the increased expression of endothelial nitric oxide synthase revealed that the activity and function of endothelial cells were enhanced. In conclusion, KNDC1 knockdown delayed endothelial cell senescence and promoted HUVEC activity and function. These results demonstrated that KNDC1 may be a novel therapeutic target for the development of agents to extend human life.

## Introduction

Cellular senescence is a process of cellular aging in which primary cells in culture lose their ability to divide (1). It has been demonstrated that senescence is associated with cellular dysfunction and occurs *in vivo* in cardiovascular diseases associated with age, such as atherosclerosis (2,3). In endothelial cells, the senescence-induced loss of replicative capacity destroys the integrity of the endothelium and impairs successful angiogenesis (4,5).

In several recent studies, protein-protein interactions have been demonstrated to be important in the molecular recognition and functional modulation of proteins in numerous signal transduction pathways (6,7). The kinase non-catalytic C-lobe domain (KIND) is a putative protein-protein interaction module (8). Four KIND-containing proteins have been reported: Spir-2 (an actin-nucleation factor), PTPN13 (a protein tyrosine phosphatase), FRMPD2 (a scaffold protein) and the Ras guanine exchange factor (RasGEF), very-KIND [v-KIND, also termed kinase noncatalytic C-lobe domain containing 1, (KNDC1)] (9,10). v-KIND, a brain-specific Ras guanine nucleotide exchange factor, has two KIND isoforms, KIND1 and KIND2, whereas the other three proteins have only one. A previous study demonstrated that v-KIND interacts with the high-molecular weight microtubule associated protein 2 (MAP2), a dendritic protein that drives negative regulation of neuronal dendrite growth. v-KIND overexpression suppresses the growth and branching of the dendrites of hippocampal neurons and cerebellar granule cells, whereas knockdown of endogenous v-KIND expression promotes dendrite growth. These findings suggest that v-KIND regulates a signaling pathway that links Ras and MAP2 to control dendrite growth (11). However, its role in vascular cell biology has not been investigated to date.

In the present study, the expression of KNDC1 with increasing age, as well as the effects of its depletion by RNA interference on senescence were investigated in human umbilical vein endothelial cells (HUVECS).

## Materials and methods

### Chemicals and reagents

Trypsin was purchased from Invitrogen Life Technologies (Beijing, China). Antibodies against extracellular signal-regulated kinase (ERK) 1/2, phospho-ERK1/2 (Thr202/Tyr204; rabbit polyclonal), p38, phospho-p38 (Thr180/Tyr182; mouse monoclonal), stress-activated protein kinase (SAPK)/c-Jun N-terminal kinase (JNK), phospho-SAPK/JNK (Thr183/Tyr185; rabbit monoclonal), endothelial nitric oxide synthase (eNOS; rabbit polyclonal), vascular cell adhesion molecule (VCAM-1; rabbit polyclonal), intercellular adhesion molecule (ICAM-1; rabbit polyclonal), p53, phospho-p53 (Ser46; rabbit polyclonal), p21 (mouse monoclonal) and p16 (rabbit polyclonal) were purchased from Cell Signaling Technology, Inc. (Cell Signaling Technology, Danvers, MA, USA). Antibodies against KNDC1 and β-actin were purchased from Santa Cruz Biotechnology, Inc. (Santa Cruz Biotechnology, Inc., Santa Cruz, CA, USA). Secondary antibodies against rabbit and mouse were purchased from Cell Signaling Technology, Inc. The pre-stained protein marker was purchased from New England Biolabs, Ltd. (Beijing, China). Luminol reagent and polyvinylidene fluoride (PVDF) membrane for western blotting were purchased from Millipore (Millipore, Billerica, MA, USA).

### Cells and cell culture

HUVECs were isolated from the umbilical cords of newborns supplied by Tongren Hospital (Beijing, China) and grown in M199 cell medium (Hyclone, Logan, UT, USA) containing 100 mg/ml streptomycin, 100 IU/ml penicillin, 40 μg/ml endothelial cell growth supplement and 20% fetal bovine serum (Hyclone) at 37°C in a humidified atmosphere of 95% air and 5% CO_2_. The cells were passaged at 80–90% confluence at a ratio of 1:2 and used for experiments at passages (P) 3–5.

### Transfections

The fourth-passage HUVECs were transfected at 70% confluence for 24 h with 20 nM small interfering (si)RNAs targeting human KNDC1 [KNDC1-siRNA1 was obtained from Santa Cruz Biotechnology, Inc. (SC-90387); KNDC1-siRNA2 was obtained from Invitrogen Life Technologies and the sense sequence was as follows: CAUCCAGGAGGAAUUUGCCUUUGAU]. A non-targeting control pool (NT-siRNA; Santa Cruz Biotechnology, Inc.) was also used. Transfections were performed using Hyperfect reagent (Qiagen, Shanghai, China) according to the manufacturer’s instructions. After 4 h, fresh medium was added and the cells were cultured for a further 72-h period prior to analysis.

### Senescence-associated β-galactosidase (SA-β-gal) staining

Endothelial cells were transfected with KNDC1-siRNA or NT-siRNA. Following incubation for 72 h, the cells were washed twice with phosphate-buffered saline (PBS) and then fixed for 5 min with PBS containing 2% formaldehyde and 0.2% glutaraldehyde. The cells were then incubated at 37°C for 10 h in a staining solution of 40 mM citric acid, sodium phosphate, pH 6.0, 1 mg/ml 5-bromo-4-chloro-3-isolyl-β-d-galactoside (X-gal; Sigma, Shanghai, China), 5 mM potassium ferrocyanide, 5 mM potassium ferricyanide, 150 mM NaCl and 2 mM MgCl_2_. SA-β-gal-positive cells were observed by microscopy (CKX31; Olympus, Beijing, China) and over 400 cells were counted in three independent fields as described previously (12).

### RNA expression analysis

Cellular RNA was extracted with TRIzol reagent (Invitrogen Life Technologies) according to the manufacturer’s instructions. RNA expression was measured by quantitative polymerase chain reaction (qPCR) with the appropriate primers using the one step SYBR PrimeScript RT-PCR kit (Takara Bio, Inc., Dalian, China). A 20 μl PCR reaction mixture was initially amplified and primer pairs for KNDC1 were obtained from Santa Cruz Biotechnology, Inc. Primer pairs for β-actin were synthesized by Shanghai Bioengineering Company (Shanghai, China). The PCR was run on an iCycler (Bio-Rad, Hercules, CA, USA). The thermal profile for SYBR qPCR was 42°C for 5 min, 95°C for 10 sec followed by 40 amplification cycles of 95°C for 5 sec and 60°C for 20 sec. Relative mRNA expression levels were calculated by the comparative cycle threshold (CT) method, using the CT values obtained for β-actin as internal references.

### Western blot analysis

Endothelial cells (7.5×10^5^) were washed with ice-cold PBS and scraped off the flask into 100 μl lysis buffer containing 25 mM Tris-HCl, pH 6.8, 1% sodium dodecylsulfate, 1 mM phenylmethylsulphonyl fluoride and protease inhibitor cocktail (Sigma). The resultant lysates were further disrupted by sonication for 10 sec at an amplitude of 35% using a VCX 500 Ultrasonic Processor (Sonics & Materials, Newtown, CT, USA) and then centrifuged at 12,000 × g for 20 min to remove particulate material. Proteins (30 μg) were fractionated by SDS-PAGE and transferred to PVDF membranes. The membranes were incubated as previously described (12).

### Cell cycle analysis

Cells (4×10^5^) were plated in 25 cm^2^ flasks and cultured in M199 medium. Following incubation for 24 h, the cells were transfected with KNDC1-siRNA or NT-siRNA. The cells were harvested at 72 h following transfection, washed twice in salt buffer (1% bovine serum albumin and 0.5% sodium azide in PBS) and fixed in 70% ethanol at 4°C overnight. After washing twice in salt buffer, the cells were stained in 50 μg/ml propidium iodide (PI) solution (Sigma) containing 100 μg/ml RNase for 1 h. Then, the cells were transferred to flow cytometry tubes with filters for cell cycle analysis.

### Capillary tube network formation

Endothelial capillary tube network formation was assessed using Matrigel (BD Biosciences, Franklin Lakes, NJ, USA). Each well of the 96-well culture plates was coated with 100 μl Matrigel and immediately incubated at 37°C for 1 h to allow gel formation. Upon gelation, transfected cells were seeded onto the Matrigel-coated 96-well plates (3×10^4^ cells/well) in 100 μl M199 medium. Following incubation for 24 h, tubule formation was analyzed under an Olympus inverted microscope (CKX31; Olympus, Beijing, China) at a magnification of ×40. Images were captured under phase contrast using an Olympus digital camera and analyzed with the Image J software (National Institutes of Health, Bethesda, MD, USA). Tubule length was determined by drawing a line along each tubule and measuring the line length in pixels. Branch points were counted manually.

### Statistical analysis

Values are presented as the mean ± standard deviation. Statistical analysis was performed using SPSS 11.0 (SPSS, Inc., Chicago, IL, USA). Results were evaluated by t-test or one-way analysis of variance followed by Bonferroni’s post-hoc tests as appropriate. A value of P<0.05 was considered to indicate a statistically significant difference.

## Results

### Senescence-like phenotype and increased transcription and expression of KNDC1 in HUVECs with increasing passage number

**Figure 1:**
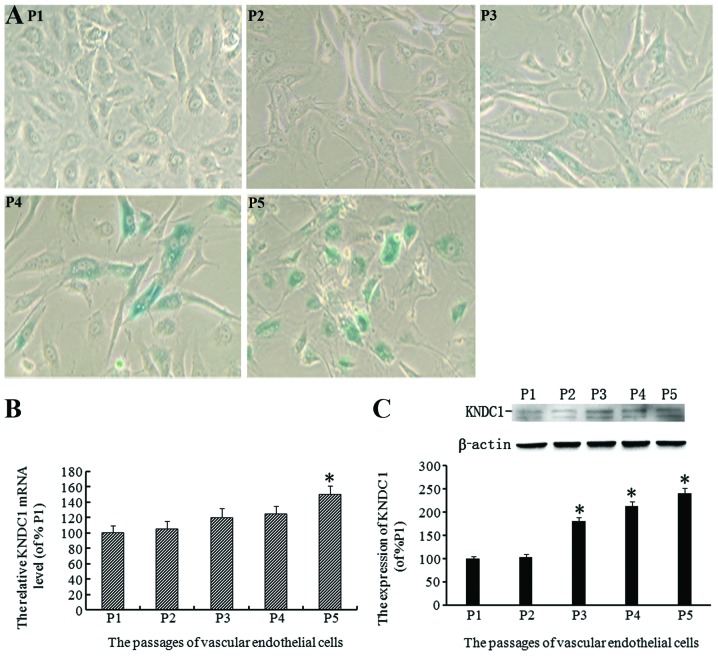
Development of a senescence-like phenotype and increased transcription and expression of KNDC1 in HUVECs with increasing passage number *in vitro*. (A) The percentage of senescent HUVECs at different passages was determined by senescence-associated β-galactosidase staining (magnification, ×200). (B) KNDC1 transcription was analyzed by quantitative polymerase chain reaction. (C) KNDC1 expression was analyzed by western blotting (n=3). Values are presented as the mean ± standard deviation. ^*^P<0.05 vs P1. KNDC1, kinase noncatalytic C-lobe domain containing 1; HUVECs, human umbilical vein endothelial cells; P, passage.

When HUVECs are cultured *in vitro*, their growth slows down and proliferation stops at the fifth passage. In the present study, it was observed that with increasing passage number, the number of aging HUVEC cells increased, as demonstrated by SA-β-gal staining. The percentages of aging cells at different passages were ~2.0±2.0% (P1), 8.0±3.0% (P2), 15.0±3.0% (P3), 35.0±6.0% (P4) and 62.0±10.0% (P5; Fig. 1A). To investigate whether KNDC1 was associated with the senescence of normal cells, the expression levels of KNDC1 in HUVECs at different passages were examined by qPCR and western blot analysis. The levels of KNDC1 mRNA exhibited a statistically significant increase in HUVECs at P1–P5 (Fig. 1B). Similarly, the levels of KNDC1 protein also demonstrated a statistically significant increase in P1–P5 HUVECs (Fig. 1C).

### Partial reversal of cellular senescence in aging HUVECs by KNDC1 knockdown

**Figure 2:**
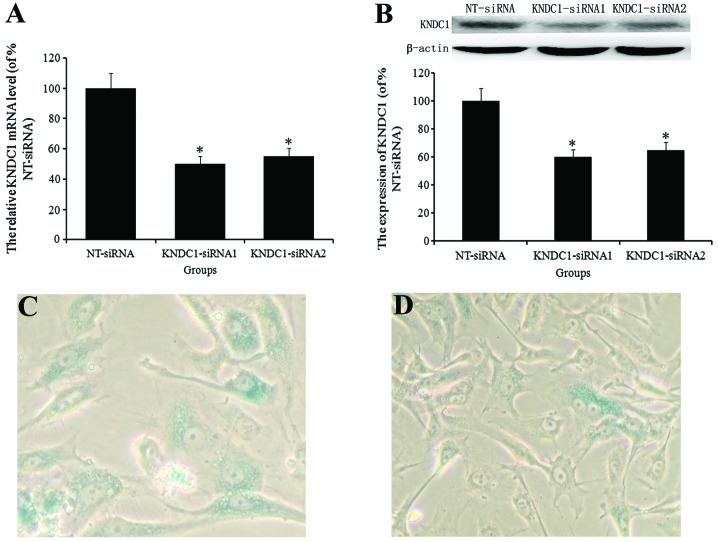
Knockdown of KNDC1 promotes cell proliferation and partially reverses cellular senescence in aging HUVECs. Following transfection with NT-siRNA or KNDC1-siRNA, the expression levels of KNDC1 mRNA and protein were determined by (A) quantitative polymerase chain reaction and (B) western blotting. The percentage of senescent HUVECs transfected with (C) NT-siRNA or (D) KNDC1-siRNA was determined by senescence-associated β-galactosidase staining (n=3; magnification, ×200). Values are presented as the mean ± standard deviation. ^*^P<0.05 vs NT-siRNA. KNDC1, kinase noncatalytic C-lobe domain containing 1; HUVECs, human umbilical vein endothelial cells; NT-siRNA, non-targeting control pool; siRNA, small interfering RNA.

Aging cells express SA-β-gal, are resistant to mitogen-induced proliferation and have a characteristically enlarged and flattened morphology. In the present study, HUVECs at P4 exhibited senescence phenotypes that distinguished them from early passage cells. To investigate the role of KNDC1 in cellular senescence, the levels of KNDC1 mRNA and protein in HUVECs at P4 were downregulated by gene silencing with KNDC1-siRNA1/2. Transfection with KNDC1-siRNA1/2 caused a ~50% decrease in KNDC1 levels (Fig. 2A and B). In subsequent studies, only KNDC1-siRNA1 was used, as KNDC1-siRNA1 and -2 had the same effect. Following repression of KNDC1 levels in HUVECs at P4, the size of the cells was similar to the size of P1 cells and the activity of SA-β-gal was decreased compared with that in NT-siRNA-transfected cells (10.0±3 vs. 38.0±7%; Fig. 2C and D).

### Knockdown of KNDC1 affects cell cycle progression

**Figure 3:**
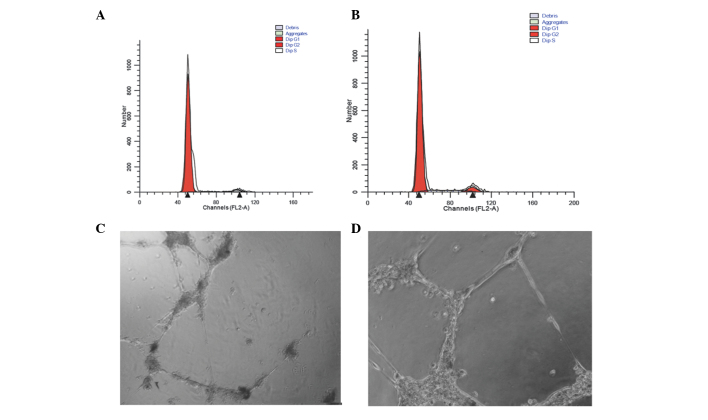
Changes in cell cycle progression and capillary tube network formation following knockdown of KNDC1. Following transfection with (A) KNDC1-siRNA or (B) NT-siRNA, the cell cycle was analyzed by flow cytometry with propidium iodide staining. HUVECs transfected with (C) KNDC1-siRNA or (D) NT-siRNA were seeded on Matrigel-coated 96-well plates (3×10^4^ cells/well) in 100 μl M199 medium. Following incubation for 24 h, the mean tubule length and the number of branch points were calculated under an Olympus inverted microscope (magnification, ×40). KNDC1, kinase noncatalytic C-lobe domain containing 1; HUVECs, human umbilical vein endothelial cells; NT-siRNA, non-targeting control pool; siRNA, small interfering RNA.

Replicatively senescent cells are known to undergo arrest in the G1/G0 phase of the cell cycle. To investigate whether cell proliferation induced by KNDC1-siRNA impacted the cell cycle, HUVECs at P4 were transfected with KNDC1-siRNA or NT-siRNA for 72 h and their DNA content was quantified by flow cytometry with PI staining. The majority of the cells transfected with NT-siRNA for 72 h had undergone G1 phase arrest, which is one of the typical phenotypes of cellular senescence. However, the number of cells in G1 phase was decreased in cells transfected with KNDC1-siRNA (Fig. 3A and B; Table I).

### Knockdown of KNDC1 improves capillary tube network formation

Since senescence is associated with impaired angiogenic function, it was examined whether KNDC1 knockdown improved the ability of HUVECs to form capillary tube networks *in vitro*. Compared with NT-siRNA-treated cells, KNDC1-silenced cells formed more developed tubule networks, in which the mean tubule length (15±5.2 mm vs. 30 mm±7.0) and the number of branches at each point (3±1.0/point vs. 2±1.0/point) increased significantly (Fig. 3C and D).

### Alterations in signaling pathways induced by knockdown of KNDC1

**Figure 4:**
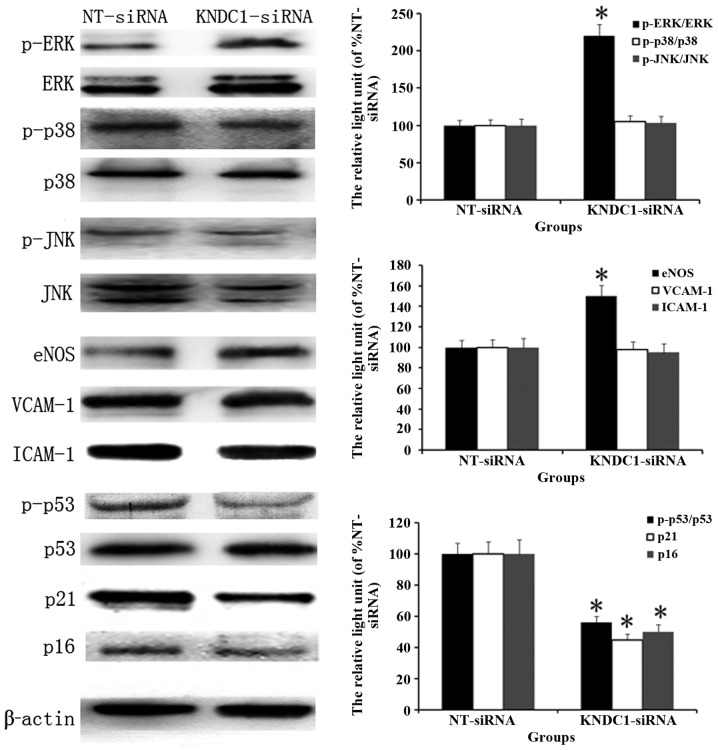
Effect of KNDC1 knockdown on signaling pathways. HUVECs were transfected with KNDC1-siRNA or NT-siRNA. Following incubation for 72 h, the expression levels of eNOS, VCAM-1, ICAM-1, p21, p16 and p-ERK, p38, JNK and p53 were examined by western blot analysis (n=3). Values are presented as the mean ± standard deviation. ^*^P<0.05 vs NT-siRNA. KNDC1, kinase noncatalytic C-lobe domain containing 1; HUVECs, human umbilical vein endothelial cells; NT-siRNA, non-targeting control pool; p, phosphorylated; siRNA, small interfering RNA; ERK, extracellular signal-regulated kinase; JNK, c-Jun N-terminal kinase; eNOS, endothelial nitric oxide synthase; VCAM-1, vascular cell adhesion molecule 1; ICAM-1, intercellular adhesion molecule 1.

In the present study, the effect of KNDC1 knockdown on the proliferation of endothelial cells [mitogen-activated protein kinase (MAPK) signaling pathway], the function of endothelial cells (eNOS, VCAM-1 and ICAM-1) and the senescence of endothelial cells (p53, p21 and p16) was investigated. It was observed that the phosphorylation of ERK, but not of p38 and JNK, was correlated to the proliferation of endothelial cells induced by the knockdown of KNDC1. The increased expression of eNOS indicated that the activity of the endothelial cells increased; however, the expression levels of VCAM-1 and ICAM-1 did not change compared with NT-siRNA-transfected control cells. In addition, the phosphorylation of p53 and the expression of p21 and p16 decreased significantly compared with NT-siRNA-transfected control cells (Fig. 4).

## Discussion

KNDC1 is a brain-specific Ras guanine nucleotide exchange factor. Overexpression of KNDC1 suppresses dendritic extension and branching in hippocampal neurons and cerebellar granule cells, whereas knockdown of endogenous KNDC1 expression promotes dendrite growth (11). However, the effect of KNDC1 on cellular senescence had yet to be elucidated. Cellular senescence, i.e., the limited ability of primary human cells to divide when cultured *in vitro*, is utilized as a model of biological aging. In common with other normal diploid cells, HUVECs have a limited capacity to divide (13). Therefore, in the present study, HUVECs were cultured *in vitro* as a model of biological aging, to investigate the effect of KNDC1 on senescence. It was observed that with increasing passages, the size of the senile HUVECs increased and the cells were positively stained with SA-β-gal. Furthermore, the transcription of the KNDC1 mRNA and expression of the KNDC1 protein increased with cellular senescence (Fig. 1). When the expression of KNDC1 was knocked down with a specific siRNA, the size and the number of the HUVECs decreased, and there was a statistically significant decrease in the number of HUVECs stained with SA-β-gal compared with control NT-siRNA-transfected HUVECs of the same passage. These results suggested that KNDC1 may be important in the progression of senescence in HUVECs and that knockdown of KNDC1 delayed this senescence. However, further investigation is required to determine whether overexpression of KNDC1 may accelerate senescence in HUVECs.

Cellular senescence is considered to be an irreversible block of cell cycle progression in populations of otherwise replication-competent cells (14,15). The proportion of arrested cells in a population rises with increasing population doublings, rather than all cells becoming senescent at once (16,17). In the present study, it was identified that knockdown of KNDC1 decreased the percentage of cells in the G0/G1 stage and increased the percentage of cells in the S and G2/M stages, compared with NT-siRNA-transfected control HUVECs from the same passage. The change in the percentage cells in G0/G1 phase was similar to the change in the expression of KNDC1.

The alterations in morphology of HUVECs transfected with KNDC1-siRNA was minimal with regard to their function. Therefore, in order to obtain further insight into the effect of KNDC1 on angiogenesis, endothelial capillary tube network formation was investigated. The results demonstrated that when HUVECs were transfected with KNDC1-siRNA, the mean tubule length and the number of branch points was significantly increased compared with HUVECs transfected with NT-siRNA. These results indicated that knockdown of KNDC1 not only delayed senescence in HUVECs but also improved endothelial capillary tube network formation.

Following this, the mechanism by which transfection with KNDC1-siRNA altered HUVEC function was investigated. The MAPK signaling pathway is associated with cell proliferation (18,19), eNOS, VCAM-1 and ICAM-1 are required for the function of HUVECs (20,21) and the p53-p21-p16 signaling pathway controls cell senescence (22,23). In the present study, it was demonstrated that the knockdown of KNDC1 promoted HUVEC proliferation via the ERK signaling pathway, but not the p38 and JNK transduction cascades. Furthermore, knockdown of KNDC1 had no effect on the expression of VCAM-1 and ICAM-1, but increased the expression of eNOS, which induces the relaxation of blood vessels. In addition, the present study provided the first evidence, to the best of our knowledge, for the involvement of KNDC1 in the senescence of human primary endothelial cells, acting through the p53 signaling pathway. It was demonstrated that knockdown of KNDC1 delayed senescence in HUVECs by decreasing the phosphorylation of p53 and the expression of the associated downstream signaling molecules, p21 and p16.

In conclusion, knockdown of KNDC1 promoted the proliferation and delayed the senescence of HUVECs. Regarding the mechanism of action, it was demonstrated that knockdown of KNDC1 increased the percentage of cells in the S and G2/M phases by inhibiting the p53-p21-p16 signaling pathway. In addition, it was observed that knockdown of KNDC1 increased the expression of eNOS and improved capillary tube network formation. Therefore, it is concluded that knockdown of KNDC1 contributed to delayed endothelial cell senescence. The results suggested that KNDC1 has the potential to be a novel target in the development of pharmacological agents to delay the aging process and extend human life.

## Figures and Tables

**Table I:** Cell cycle distribution of P4 generation HUVECs following KDNC1-siRNA treatment for 72 h (n=3).

	Percentage (%)		
	
Groups	G1	S	G2/M
NT-siRNA	94.25±5.6	4.75±1.0	1.00±0.5
KNDC1-siRNA	85.78±4.5*	8.29±1.0*	5.93±1.0*

Data are presented as the mean ± standard deviation.

* P<0.05 compared with NT-siRNA.

KNDC1, kinase noncatalytic C-lobe domain containing 1; HUVECs, human umbilical vein endothelial cells; P4, passage 4; NT-siRNA, non-targeting control pool; siRNA, small interfering RNA.
